# Fast automatic quantitative cell replication with fluorescent live cell imaging

**DOI:** 10.1186/1471-2105-13-21

**Published:** 2012-01-31

**Authors:** Ching-Wei Wang

**Affiliations:** 1 Graduate Institute of Biomedical Engineering, National Taiwan University of Science and Technology, Taipei, Taiwan

## Abstract

**Background:**

live cell imaging is a useful tool to monitor cellular activities in living systems. It is often necessary in cancer research or experimental research to quantify the dividing capabilities of cells or the cell proliferation level when investigating manipulations of the cells or their environment. Manual quantification of fluorescence microscopic image is difficult because human is neither sensitive to fine differences in color intensity nor effective to count and average fluorescence level among cells. However, auto-quantification is not a straightforward problem to solve. As the sampling location of the microscopy changes, the amount of cells in individual microscopic images varies, which makes simple measurement methods such as the sum of stain intensity values or the total number of positive stain within each image inapplicable. Thus, automated quantification with robust cell segmentation techniques is required.

**Results:**

An automated quantification system with robust cell segmentation technique are presented. The experimental results in application to monitor cellular replication activities show that the quantitative score is promising to represent the cell replication level, and scores for images from different cell replication groups are demonstrated to be statistically significantly different using ANOVA, LSD and Tukey HSD tests (*p-value *< 0.01). In addition, the technique is fast and takes less than 0.5 second for high resolution microscopic images (with image dimension 2560 × 1920).

**Conclusion:**

A robust automated quantification method of live cell imaging is built to measure the cell replication level, providing a robust quantitative analysis system in fluorescent live cell imaging. In addition, the presented unsupervised entropy based cell segmentation for live cell images is demonstrated to be also applicable for nuclear segmentation of IHC tissue images.

## 1 Background

**Figure 1:**
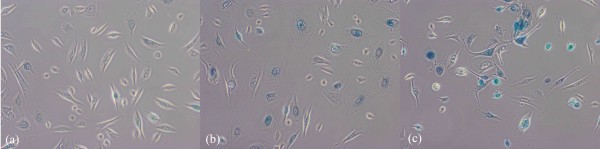
**Negative correlation between cell replication degree and stain level**. We observed that Live cell imaging reveals cell replication degree by negative correlation with stain level: (a) high cell replication with low stain for the Scr samples, (b) medium cell replication activity with mediumn stain for BRCA1 samples, (c) low cell replication with high staining level for p63 samples.

Live cell imaging is an useful tool to monitor cellular activities in living systems and to study complex biological processes in great detail [1]. In recent years, technological advances include sensor sensitivity, computing power, brighter and more-stable fluorescent proteins, but expertise in the automated image analysis is required to harness the full potential that live-cell microscopy offers. Kitamura et al. [2] showed that live cell imaging reveals replication of individual replicons in eukaryotic replication factories, using time-lapse microscopy. In our investigation on BRCA1 [3], p63 [4] and Scr [5] in breast cancer, a negative correlation was discovered by manual observation in live cell imaging between the cell replication activities and stain expression level using fluorescence microscopy (Figure 1). That is the higher degree of blue stain appears, the less cell replication activity occurs. (More information on the associated biological study has been published in [6].)

However, manual quantification is subjective and results tend to be poorly reproducible. Worse, the ability of manual quantification is limited as human eye is not sensitive to fine differences in color intensity, and only restricted semi-quantitative mode can be provided. As a result, an automated quantification approach of live cell imaging is needed for monitoring cell proliferation activities.

Auto-quantification of live cell imaging is not a straightforward problem to solve. As the sampling location of the microscopy changes, the amount of cells captured within each image varies, which makes simple measurement methods such as the sum of blue intensity values or the total number of positive blue stain within images inapplicable. Scores by such simple quantification models can vary a lot according to the camera sampling location, and worse images with low quantity of cells and high staining level can have similar scores with images with high quantity of cells but medium staining level. Hence, cell segmentation is required to identify regions of interest prior to generate a robust measurement of the fluorescence degree of cell expression, D. The quantification of fluorescent degree can be formulated as follows.

(1) $$D=\overset{n}{\underset{i=1}{\sum }}\left(\underset{j\subset A}{\sum }f_{j}/\#A\right)/n$$

where n is the number of cells, A is the set of cell locations, and *f*_*j *_is the fluorescence level at location j.

**Figure 2:**
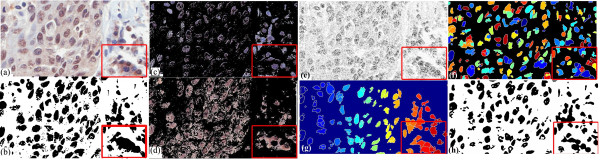
**Performance comparison with K-means clustering, Otsu clustering, the proposed entropy-based method**. (a) an IHC Lung carcinoma tissue image, (b) poor segmentation by Otsu unsupervised clustering, which automatically separates the image into two classes but contains a lot of false detection, (c)(d) poor segmentation by K-means clustering, which automatically separates the image into three classes here but the resulting clusters are poor in nuclear segmentation, (e) over-segmentation by Vincent-Soille watershed algorithm [12] (f) poor segmentation result by marker-controlled watershed method [14] (g) segmentation result with many false positives by optimized watershed transformation (adapted from [13]) (h) improved nuclear segmentation by the proposed entropy-based method.

Accurate segmentation is a crucial step for the quantitative microscopic image analysis. In biomarker analysis, accurate segmentation of nuclei is an important step for the quantitative immunohistochemistry (IHC) image analysis of nuclear malignancy; among the most useful features for cytological applications have been measures of nuclear size, pleomorphism and chromatin appearance [7]. To evaluate and analyze the properties of nuclei, segmentation of nuclear regions are needed. However, accurate segmentation of nuclei is often difficult because of the heterogeneous cellular staining and nuclear overlapping. The simplest approach for segmenting nuclei is a global thresholding, which is adjusted manually or determined by the measurement of the image histogram. Such method works well in high-contrast-feature tissue images such as applications to measure oestrogen and progesterone receptor levels in breast cancer [8], but is not suitable for tissue images with varying image features (in Figure 2(a), some nuclei appear distinct but in the highlighted region, the contrast of image features is low). Mao et al. [9] presented a supervised learning image segmentation method for P53 IHC images by separating two classes of image pixels (background and nuclei) from color image, using the learnt transformation formula from the dataset of background and nuclei pixels of the studied images, and thresholding the extracted nuclear image pixels using otsu clustering. Adaptive local thresholding techniques, which utilize local content information and automatically separates image pixels into different classes, produce significantly better results in comparison to the global thresholding method; we tested two commonly adopted techniques (Otsu clustering [10] and K-means clustering [11]), showing that the unsupervised local thresholding methods are still not sufficient for nuclear segmentation (Figure 2(b,c,d)).

Another popular approaches for nuclear detection are watershed algorithms [12]. As in practice, the Vincent-Soille watershed tends to produce an over-segmentation(Figure 2(e)), we tested a watershed algorithm (adapted from [13]) and marker-controlled watershed method [14] with optimized empirically-set parameters (Figure 2(g,f)). However, the watershed methods still produce many false positives and false negatives. As a result, a robust method for nuclear segmentation in challenging IHC tissue images is needed, and in this study, an entropy-based method is presented, which can also be extended for nuclear segmentation in IHC tissue images (an output by the proposed method is shown in Figure 2(h)).

**Figure 3:**
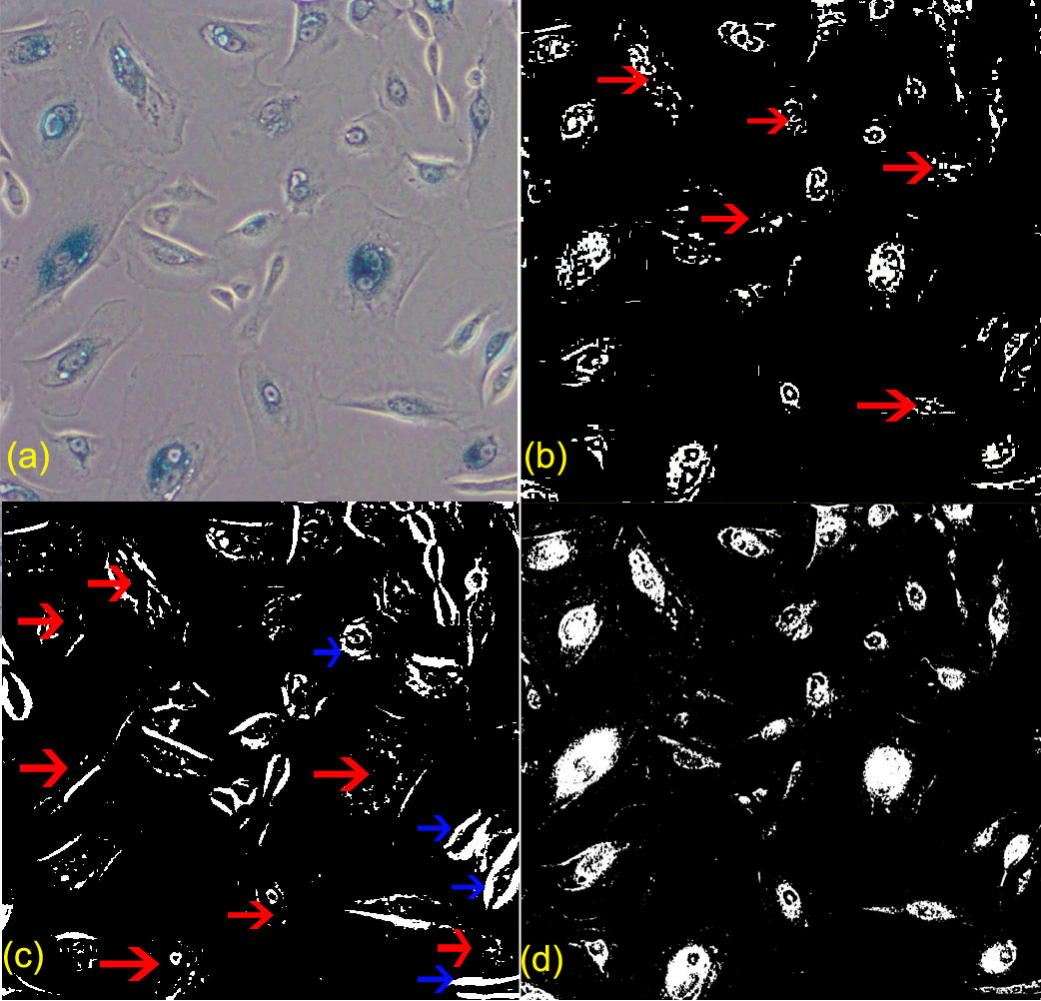
**Comparison of automatic cell segmentation results by K-means clustering, Otsu clustering and a proposed method**. Comparison of cell segmentation results, with the segmented regions by individual techniques highlighted in white: (a) raw image, (b) cell segmentation by K-means clustering with various misdetections pointed by the red arrows, (c) cell segmentation by Otsu clustering with plenty misdetections pointed by the red arrows and false detection pointed by the blue arrows (d) cell segmentation by the presented method with greatly improved outcome (much less misdetection and false detection).

In live cell images, two commonly adopted techniques (Otsu clustering [10] and K-means clustering [11]) were tested, showing that the unsupervised local thresholding methods are still not sufficient for cell segmentation on the fluorescent images. Figure 3 compares the cell segmentation results by Otsu clustering in (c), which contains lots of misdetection and false positives, by K-means clustering in (b), which still contains some misdetection and by the presented method in (d), which contains much less misdetection and false positives. To sum up, conventional Otsu clustering and K-means clustering are poor in segmentation of the IHC tissue images and high resolution fluorescent live cell images as these methods suffer from serious local variations and produce poor segmentation results.

In this paper, an entropy based cell segmentation method is developed for fluorescent microscopic images and an automated quantification system of live cell imaging is built to analyze the cell replication level. The method is invariant to the camera sampling location and the amount of cells appearing in the image. In addition, it takes less than 0.5 second to process each image with dimension 2560 × 1920.

## 2 Results and Discussion

In evaluation, the quantification scores were tested with one-way ANOVA, Tukey HSD and Least square difference (LSD) using SPSS software [15]. In Table 1, the overall one-way ANOVA results are significant, indicating that the mean scores of three groups are significantly different. Next, multiple post hoc comparisons were conducted to compare mean difference between two groups. In Table 2, the results by both Tukey HSD and LSD tests show that the mean differences between any two groups are statistically significant (*p-values *< 0.01). To sum up, the tests show that the quantitative cell replication score is distinctive and varies according to the cell replication level.

**Table 1:** ANOVA Test on the quantification score

	sum of squares	df	mean square	F	Significance
Between groups	1429.6	2	714.8	43.754	< 0.001
Within groups	98.022	6	16.337		
Total	1527.622	8			

The overall one-way ANOVA results show that the mean difference is significant, indicating that the mean scores of three groups are significantly different.

The test shows that the quantitative cell replication score is distinctive and varies according to the cell replication level in our experiments.

**Table 2:** Multiple Comparison using Tukey HSD and LSD tests

	Dataset (I)	Dataset (J)	mean diff. (I-J)	std. error	Significance
Tukey HSD	p63	BRCA1	-15.29	3.3	0.008*
		Scr	-30.87	3.3	< 0.001*
	BRCA1	p63	15.29	3.3	0.008
		Scr	-15.579	3.3	0.008*
	Scr	p63	30.87	3.3	< 0.001*
		BRCA1	15.58	3.3	0.008*

LSD	p63	BRCA1	-15.29	3.3	0.004*
		Scr	-30.87	3.3	< 0.001*
	BRCA1	p63	15.29	3.3	0.004
		Scr	-15.579	3.3	0.003*
	Scr	p63	30.87	3.3	< 0.001*
		BRCA1	15.58	3.3	0.003*

Multiple post hoc comparisons were conducted to compare mean difference between two groups. Results of both Tukey HSD and LSD tests show that the mean differences between any two groups are statistically significant (*p - values *< 0.01). This test further confirms that the quantitative cell replication score is distinctive and varies according to the cell replication level.

**Figure 4:**
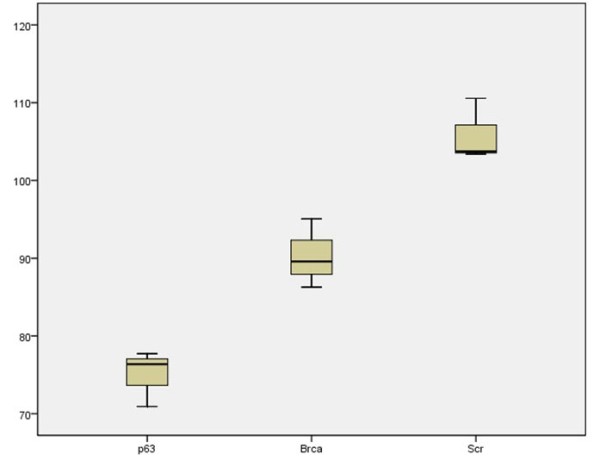
**Consistent quantification results in variant to the number of cells appearing**. The proposed cell replication scores are stable with consistent outputs for images in the same cell replication level, invariant to the number of cells captured in the image: three live cell images from individual cell replication group were selected; the first column contain low quantity of cells and the second and third columns contain high quantity of cells. The scores for each row are consistent. (a,b,c) from the low cell replication group (p63), (d,e,f) from the medium cell replication group (BRCA1), (g,h,i) from the high cell replication group (Scr).

**Figure 5:**
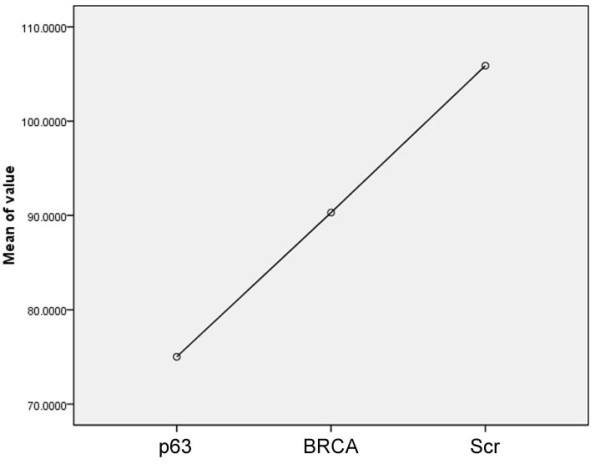
**Means Plot of Quantification Scores**. This figure displays the means plot of the three group from the low cell replication group (p63) to the high cell replication group (Scr).

Furthermore, the quantification score is stable and consistent, invariant to the amount of cells appearing in individual images. Figure 4 displays images from the three different cell replication groups (row 1 for p63; row 2 for Brca; row 3 for Scr), showing that scores for images containing a large number of cells or comparably low number of cells are consistent and distinct among different groups. In addition, a means plot generated by SPSS software is illustrated in Figure 5. Regarding the system processing speed, the imaging system is implemented in java, and without code optimization, it takes less than 0.5 second for each image on a standard PC with CPU 3.16 GHz.

### 2.1 Results of the extension to IHC tissue images

**Figure 6:**
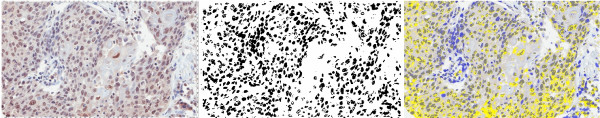
**Nuclear segmentation in IHC tissue image analysis**. Extracted nuclei (middle) can be used for quantitative IHC analysis such as the percentage or intensity levels of positively stained nuclei (right), where gray areas are positively nuclei and blue areas are negatively stained nuclei.

An extended version of the proposed entropy-based segmentation technique is built for nuclear segmentation of IHC images. A quantitative performance evaluation was conducted by comparing the ground truth data and the system output. In pixel-based quantitative performance evaluation, the system achieves 92% precision and 75% recall rates and has been demonstrated to be promising in nuclear cell detection on lung tissue images. In biomarker discovery applications, the extracted regions of nuclei can then be used to analyze nuclei malignancy; it can be used to quantify the percentage or intensity levels of positively stained nuclei as shown in Figure 6.

## 3 Conclusions

It is often necessary in experimental research to quantify the dividing capabilities of cells when investigating manipulations of the cells or their environment. The presented technique provides a new type of information in monitoring cell replication activity and greatly empowers live cell imaging in studying cellular process. The availability of this novel technique should facilitate a more precise and comprehensive evaluation of cell proliferation and aid in the interpretation of results.

**Figure 7:**
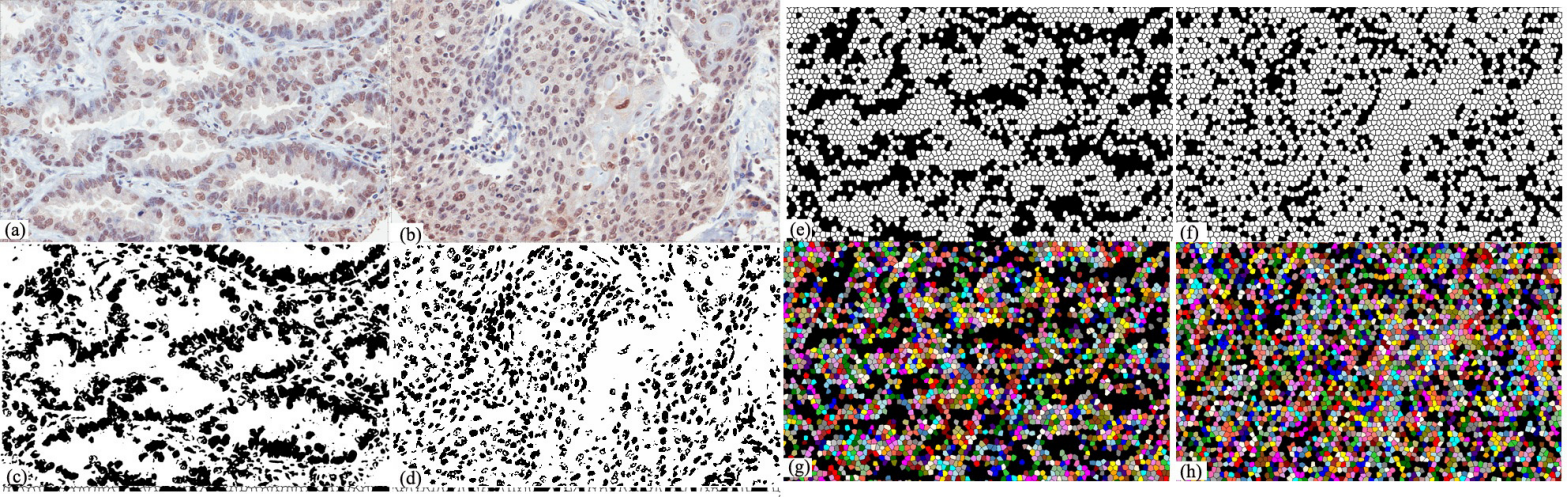
**Cancer subtype classification based on nuclear layout patterns**. (a) adenocarcinoma tissue image with snake-like glandular structure, (b) squamous carcinoma tissue image with sheet-like structure, (c,d) extract nuclei architecture, (e,f) scene abstraction to remove isolated or small islands of nuclei, (g,h) detect connecting components and look for large regions of connecting components as glandular structure for pattern recognition of adenocarcinoma.

In addition, we have presented an unsupervised entropy-based system to detect nuclei in IHC lung tissue slides. The method has shown to perform well in image segmentation in the experiments. Furthermore, the extracted nuclei information is demonstrated to be useful in quantitative IHC. In addition, apart from analyzing nuclei activity, we would like to extend the method for cancer subtypes classification in lung cancer. We plan to utilize the identified nuclei architecture information for automated classification of cancer subtypes. Figure 7 shows two types of lung carcinomas, including adenocarcinoma and squamous carcinoma. The characteristic histologic feature of Adenocarcinoma is glandular structure (Figure 7(a)), where nuclei form snake-like shapes. On the other hand, the characteristic feature of squamous carcinoma(Figure 7(b)) is sheet-like structure. Hence, after obtaining nuclei architecture information (Figure 7(c,d)) by the proposed method, scene abstraction function can be applied (Figure 7(e,f)) to remove isolated or small islands of nuclei. In future work, we plan to utilize the extracted connecting components (Figure 7(g,h)) as patterns to distinguish the two non-small cell lung cancers by detecting large regions of connecting components as glandular structures and recognizing the tissue slides as adenocarcinoma cases.

## 4 Methods

### 4.1 Materials in fluorescence microscopy images

**Figure 8:**
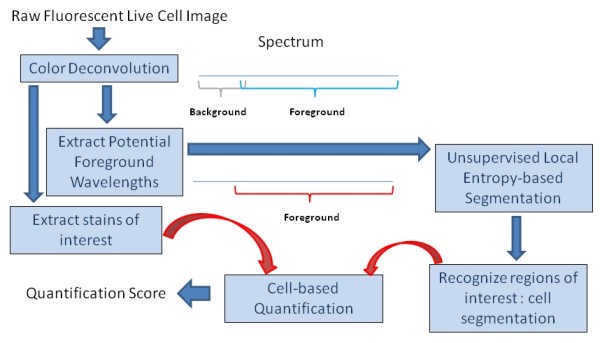
**System framework**. An overview of the presented automatic quantitative method is illustrated. The algorithm contains three main parts, including a color deconvolution algorithm to extract the potential foreground wavelength and extract the stains of interest, an unsupervised local entropy-based segmentation to identify the areas where cells locate, and a cell-based quantification function to measure the level of the stain of interest within the cell regions.

Nikon TiS inverted fluorescence microscopy is used to capture live cell images. The image dimension of each image is 2560 × 1920 with file size 14.1 megabytes. Three breast tumour samples stained with different proteins were used to generate three representative data sets, which cover a spectrum of cell replication levels; samples stained with p63 represent the group with low cell replication activity, samples stained with BRCA1 represent mediumn cell replication group, and samples stained with Scr represent the group with highly active cell replication activity. For each cell sample, twenty live cell images were captured, and from each image set, three images were manually selected to represent a spectrum of cell quantity level (high, medium and low). The presented method was tested on these images of each group to generate quantification scores, and the distribution of the output scores by the presented method is illustrated in Figure 8.

### 4.2 Materials in immunohistochemistry tissue images

An extended version of the proposed entropy-based segmentation technique is built for nuclear segmentation of IHC images. A tissue microarray (TMA) slide was scanned using Aperio Scanscope CS2 (Aperio Technologies Inc. San Diego USA), at 40x objective magnification. Nine different tissue cores were randomly selected for evaluation; the image size of individual tissue cores is around 2896 × 2756 and the nuclear areas of each tissue core were manually marked to produce ground truth data. A quantitative performance evaluation was conducted by comparing the ground truth data and the system output.

### 4.3 Automatic cell based quantification approach in fluorescent microscopic images

In general image data, the color of the image pixels represents the appearance of the surface of an object. This however is not always applicable to the microscopic images. In the fluorescent microscopic images, the gray color of the background regions can not represent the true appearance of specimens. These background areas are in fact transparent and become gray when captured using a digital microscopic system. Hence, segmentation by general unsupervised clustering techniques, such as Otsu or K-means, based on the raw digital image appearance information does not produce good segmentation results because those information can be misleading.

**Figure 9:**
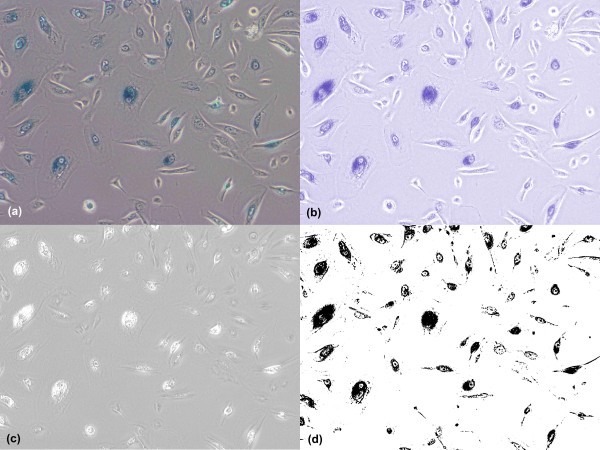
**Color deconvolution**. Color separation for foreground cells and background color and cell segmentation based on the extracted color information: color deconvolution is applied to separate the blue stain (b) and background color (c). The extracted blue color is further applied with multistage entropy-based segmentation to segment the cell area (d) for quantification.

As in the background empty regions, all excitation light can be reflected and the emission light can all pass through, the non-stained empty background regions therefore contains different wavelength from the foreground cell regions, and the stained specimen components show different wavelengths (with broad spectrum) from the background regions. Therefore, a method is built to extract possible foreground specimen wavelengths first, and then utilizes this true appearance information for further cell segmentation using a localized entropy-based segmentation approach. Then, a quantification system is built to test the method. The framework of the quantification system is illustrated in Figure 9.

#### 4.3.1 Extraction of Foreground Color Information

##### Color representation

The Lambert-Beer's law describes an exponential relationship between the intensity of monochromatic light transmitted through a specimen and the amount of stain present in the specimen:

(2) $$I_{1}\left(\lambda \right)=I_{0}\left(\lambda \right)\text{exp}\left(-\alpha \cdot c\left(\lambda \right)\right)$$

where *I*_1_(λ) is the intensity of light of wavelength λ transmitted through the specimen (the intensity of light detected), *I*_0_(*λ*) is the intensity of light of wavelength λ entering the specimen, *α *is the amount of stain per unit area of the specimen, and *c*(λ) is a wavelength-dependent factor reflecting the absorption characteristics of the particular stain.

The CCD RGB cameras use three broad-band filters to capture color images in three channels. As the relative intensity *I*_*r*_, *I*_*g*_, *I*_*b *_in each of the RGB channels depends on the concentration of stain in a nonlinear way [16], the intensity values of the image can not directly be used for separation and measurement of each of the stains, but the optical density (OD) for each channel can be defined as

(3) $$D=\text{ln}\left(\frac{I_{1}}{I_{0}}\right)=\alpha \cdot c$$

The OD for each channel is linear with the amount of stain, given the absorption value, and can therefore be used for extracting the amount of stain in a specimen. Each stain can be characterized by a specific OD for the light in each of the three RGB channels, which can be represented by a 3 × 1 OD vector describing the stain in the OD-converted RGB color space [17]. Hence, in the case of two stains, the color system can be described as

(4) $$\left(\begin{array}{ccc} I_{r1} & I_{g1} & I_{b1}\\ I_{r2} & I_{g2} & I_{b2} \end{array}\right)$$

where each row represents a specific stain and each column represents the OD as detected by RGB channels for individual stain.

Color deconvolution [17] can be used to obtain independent information about each stain's contribution based on orthonormal transformation of the RGB information, and the transformation has to be normalized to achieve correct balancing of the absorbtion factor for separate stains. For normalization, each OD vector is divided by its total length to obtain a normalized OD array *A*. If *C *is the 2 × 1 vector for amounts of the two stains at a particular pixel, then the vector of OD levels detected at that pixel is *D *= *CA*. Defining *K *= *A*^-1 ^as the color-deconvolution array, we can therefore obtain individual stain information by *C *= *KD*.

**Figure 10:**
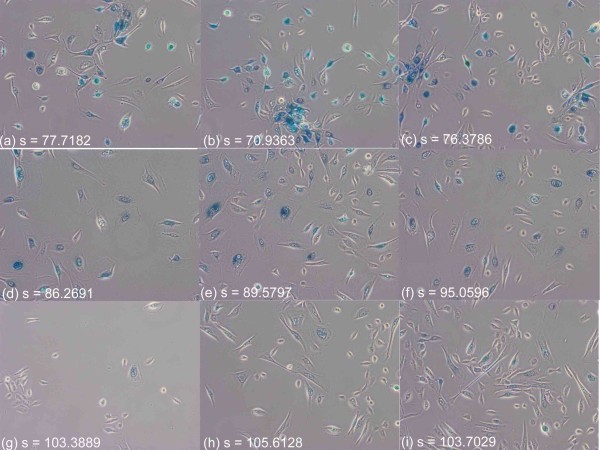
**Positive correlation between the quantitative results by the presented technique and the actual cell replication level**. The distribution of the quantitative cell replication scores by the proposed technique is positively correlated to the actual cell replication level. Moreover, each score sub-distribution of each group is distinctive as there is no overlap of score distribution between any two groups with different cell replication level. Table 2 also proves that scores of different groups are statistically significantly different.

In this study, color deconvolution is used to separate the potential single stain channel information from the background color channel, and the color-deconvolution array is defined as:

(5) $$K=\left(\begin{array}{ccc} 0.6443 & 0.7167 & 0.2669\\ 0.1754 & 0.9723 & 0.1546 \end{array}\right)$$

where the first row vector is used to compute the blue stain channel information (Figure 10(b)), *I*_*B*_, and the second row is for the background color channel information (Figure 10(c)). Cell segmentation (section 4.3.2) is based on the extracted blue color information to identify the foreground cell (Figure 10(d)) for quantification.

#### 4.3.2 Local Entropy-based Cell Segmentation

Given the extracted color image, *I*_*B*_, the cell is segmented by local entropy-based segmentation. According to Shannon's theorem [18], if the event *i *occurs from a set of valid events with the probability *p*_*i*_, the amount of uncertainty related to the event is equal to *H*_*i *_= -log(*p*_*i*_)(*bits/symbol*), and the amount of the uncertainty that the source of the events generates is equal to *H *= - Σ (*p*_*i *_log(*p*_*i*_))(*bits*). The idea behind local entropy method is to divide the processed image into separate regions and then to analyze each region separately as information source.

Therefore, we separate the foreground cell and background as two different information sources, by searching the maximum local entropy to obtain the cut-off point. Given the input color information, we first compute the normalized image histogram information, $P=\left\{p_{0},...,p_{2^{c}-1}\right\}$ where the valid intensity scales from 0 to 2^*c *^- 1. Then, the image entropy E(P) is calculated using discrete histogram P as follows.

(6) $$E\left(P\right)=H\left(P\right)=\left\{H_{0}...H_{2^{c}-1}\right\}$$

where

(7) $$H\left(A\right)=-\overset{j}{\underset{i=0}{\sum }}p_{i}\text{log}p_{i}$$

(8) $$H\left(B\right)=-\overset{2^{c}-1}{\underset{i=j}{\sum }}p_{i}\text{log}p_{i}$$

(9) $$H_{j}=-\text{log}P\left(A\right)-\text{log}P\left(B\right)-\frac{H\left(A\right)}{P\left(A\right)}-\frac{H\left(B\right)}{P\left(B\right)}$$

where *j *∈ {0...2^*c *^- 1}, *A *= {0...*j*} and *B *= {2^*c *^- 1...*j*}.

The entropy maximum is calculated as *E*_*max*_(*P*) = max *H*(*P*), which defines the cut-off point *j *for assigning image pixels into different classes where $H\left(P\right)=\left\{H_{0}...H_{2^{c}-1}\right\}$. Here we compute the optimal cut-off point and categorize pixels of an input image into 2 classes (Figure 10(d)), including foreground cells and background.

(10) $$j^{*}=\text{arg max}H\left(P\right)$$

(11) $$I^{*}\left(p_{i}\right)=\left\{\begin{array}{cc} 0 & i\leq j^{*}\\ 2^{c}-1 & i>j^{*} \end{array}\right)$$

where *i *∈ {0...2^*c *^- 1}.

#### 4.3.3 Quantification Function

Given the blue stain channel information *I*_*B*_(*X*,*Y*) and segmented image I*(*X*,*Y*), the cell replication quantification score is formulated as follows.

(12) $$s=\frac{\sum_{\left(m,n\right)}I_{B}(m,n)}{\left|I^{*}(m,n)\right|}|{}_{(m,n)\in (X,Y)\wedge I^{*}(m,n)=0}$$

### 4.4 Software

The developed software is platform independent and thus can be executed in different operation systems such as Windows, Linux or Mac. The software with some test images can be downloaded from the author's website (http://www-o.ntust.edu.tw/~cweiwang/Cell/).

### 4.5 Extension to IHC tissue images

An extended version of the proposed entropy-based segmentation technique is built for nuclear segmentation of IHC images. Given an IHC image, we first separate independent DAB and Haematoxylin stain contributions by the color deconvolution approach [17], and assign a normalized optical density (OD) matrix, *M*, to describe the colour system for orthonormal transformation as follows:

(13) $$M=\left(\begin{array}{ccc} R & G & B\\ 0.65 & 0.704 & 0.286\\ 0.072 & 0.99 & 0.105\\ 0.268 & 0.57 & 0.776 \end{array}\left|\begin{array}{c} \text{Haematoxylin}\\ \text{Eosin}\\ \text{DAB} \end{array}\right)\right)$$

Next, a multistage entropy-based segmentation of nuclei is applied. After calculating 2D image histogram entropy function, we first apply an eight stage maximum entropy function to automatically separate input image into eight layers, and then a two stage entropy function to extract potential regions of nuclei, which is then processed by morphological operations to produce final nuclear segmentation results. The algorithm is described below.

• divide histogram into four equal sub-histograms *P*_1_, *P*_2_, *P*_3_, *P*_4_, obtaining *j*_1_, *j*_3_, *j*_5 _where *j *∈ 0...2^*c *^- 1

• compute maximum entropy points *j*_0_, *j*_2_, *j*_4_, *j*_6 _for the four different *P *intervals, where *j*_0 _= arg max *H*(*P*_1_), *j*_2 _= arg max *H*(*P*_2_), *j*_4 _= arg max *H*(*P*_3_), *j*_6 _= arg max *H*(*P*_4_).

• use *j*_0_...*j*_6 _to categorize input image into eight layers

• calculate new histogram P*

• compute j* = arg max *H*(P*) and categorize input image into 2 categories, including nuclei and non-nuclei.

• apply the morphological operations described below

The purpose of the morphological function is both to reduce spurious false positive detection and increase low contrast true negative detection. The method re-assigns each image pixel value using the most frequent intensity level within its neighborhood. Given an image *I*(*X, Y*) and neighborhood radius *r*, the output image *I*'(*X, Y*) is formulated as follows.

(14) $$I^{\prime }\left(x,y\right)=\text{arg}\underset{I}{\text{max}}\left(\#I\left(K,L\right)\right)$$

where *K *= {*x *- *r*,..., *x *+ *r*}, *L *= {*y *- *r*,..., *y *+ *r*}, and r is empirically set as 3.

## 5 Competing interests

The author declares that there is no competing interest.

## 6 Authors' contributions

CW carried out the design of the study, built the method and performed the statistical analysis. The author has read and approved the final manuscript.
